# Muscle shear wave elastography, conventional B mode and power doppler ultrasonography in healthy adults and patients with autoimmune inflammatory myopathies: a pilot cross-sectional study

**DOI:** 10.1186/s12891-021-04424-0

**Published:** 2021-06-12

**Authors:** Shereen Paramalingam, Merrilee Needham, Warren Raymond, Frank Mastaglia, Daniel Lightowler, Narelle Morin, Peter Counsel, Helen Isobel Keen

**Affiliations:** 1 University of Notre Dame Australia, Fremantle, Western Australia Australia; 2 Department of Rheumatology, Fiona Stanley Hospital, 11 Robin Warren Dr, Western Australia 6150 Murdoch, Australia; 3 Institute of Immunology and Infectious Diseases, Murdoch University, Murdoch, Western Australia Australia; 4 Department of Neurology, Fiona Stanley Hospital, Murdoch, Western Australia Australia; 5 Perron Institute for Neurological and Translational Science, Nedlands, Western Australia, Australia; 6 University of Western Australia, Crawley, Western Australia Australia; 7 Sonowest Healthcare, Siemens Healthineers, Bayswater, Australia; 8 Department of Radiology, Perth Children’s Hospital, Nedlands, Western Australia Australia

**Keywords:** Shear wave elastography, ultrasound, idiopathic inflammatory myopathy, myositis, imaging

## Abstract

**Background:**

Before the role of shear wave elastography (SWE) and B mode ultrasound (US) in the diagnosis of different forms of idiopathic inflammatory myopathies (IIM) can be investigated, normative data is required. This study aimed to describe and then compare normative SWE and B mode ultrasound metrics of muscles in healthy controls and patients with IIM.

**Methods:**

Twenty nine healthy adult controls and 10 IIM patients (5 with inclusion body myositis and 5 with necrotising autoimmune myopathy) underwent a full clinical examination, laboratory investigations, SWE and US measurements of selected proximal and distal limb muscles. Shear wave speed (SWS) and multiple US domains [echogenicity, fascial thickness, muscle bulk and power Doppler (PD)] were measured in both groups.

**Results:**

In healthy controls (n = 29; mean age 46.60 ± 16.10; 44.8 % female), age was inversely correlated with SWS at the deltoid (stretch) (Rs. -0.40, p = 0.030) and PD score at the deltoid (rest) (Rs. -0.40, P = 0.032). Those ≥ 50 years old had a lower SWS at the deltoid (stretch) compared to the < 50 year group (2.92 m/s vs. 2.40 m/s, P = 0.032). Age correlated with increased echogenicity in the flexor digitorum profundus (Rs. 0.38, P = 0.045). Females had a smaller muscle bulk in the deltoid (P = 0.022). Body mass index (BMI) was inversely associated with SWS in the deltoid (stretch) (Rs – 0.45, P = 0.026), and positively correlated with echogenicity in the deltoid (Rs. 0.69, P = 0.026). In patients ≥50 years of age, patients with IIM (mean age 61.00 ± 8.18; females 20.0 %) had a higher proportion of abnormal echogenicity scores at the flexor digitorum profundus (FDP) (40.00 % vs. 14.30 %, P = 0.022) and tibialis anterior (TA) (80.00 % vs. 28.60 %, P = 0.004). Fascial thickness was lower in the FDP (0.63mm vs. 0.50mm, p = 0.012) and TA (0.58mm vs. 0.45mm, P = 0.001).

**Conclusions:**

Our findings suggest there is scope for US techniques to be useful for diagnostic screening of affected muscles in patients with IIM, especially in those with suspected inclusion body myositis or necrotising autoimmune myopathy. We provide normative data for future studies into SWE and US techniques in skeletal muscle. The differences between IIM patients and controls warrant further study in a broader IIM patient cohort.

**Supplementary Information:**

The online version contains supplementary material available at 10.1186/s12891-021-04424-0.

## Background

Idiopathic inflammatory myopathies (IIM) are a rare group of skeletal muscle diseases, unified by immune-mediated muscle damage, that result in muscle fibre loss and weakness. Subtypes of IIM include dermatomyositis (DM), polymyositis (PM), necrotising autoimmune myopathy (NAM), overlap myositis (OM) and inclusion body myositis (IBM) [1]. The subtypes of IIM are heterogeneous with clinically distinct presentations and patterns of muscle involvement, serum autoantibody profiles, and response to therapies. Despite being associated with high morbidity, and increased mortality [2, 3], most subtypes of IIM are treatable; with earlier diagnosis and management usually translating to better health-related outcomes [4].

Currently, muscle biopsy remains the gold standard for confirming the diagnosis of IIM and to exclude other muscle disorders. However, delayed or misdirected biopsies contribute to the 10–45 % false-negative biopsy rate [5]. Increasingly, magnetic resonance imaging (MRI) is used as a screening procedure for the presence and extent of muscle abnormalities, such as inflammation, oedema, fatty infiltration, and muscle atrophy [6]; and may help in determining the optimal site to biopsy [5]. However, the feasibility of MRI is limited by expense, accessibility and patient tolerability.

Point-of-care imaging tools such as shear wave elastography (SWE) and ultrasound (B mode and power Doppler) are being studied to assess their utility in the timelier diagnosis and monitoring of patients with IIM [7–11]. Although these modalities have been evaluated in neuromuscular disease and muscle injury [12], their use in IIM remains understudied, particularly in inclusion body myositis (IBM) and necrotising autoimmune myopathy (NAM) that pose diagnostic and management difficulties for clinicians [13, 14]. Moreover, there are a limited number of studies with normative ultrasound data for muscle groups that are typically most affected by these forms of myopathy[15, 16].

Shear wave elastography (SWE) is a novel ultrasound technique which uses a modified low frequency/high-intensity B mode US push-pulse called, acoustic radiation force impulse (ARFI) to displace tissue and determine muscle stiffness [17, 18]. Muscle stiffness is recorded as Shear Wave Speed (SWS) in m/s, or as Muscle Shear Modulus (MSM), in kilopascals (kPa). Lower SWS is associated with lower muscle stiffness (10), and SWE has shown age-related differences in muscle stiffness which correlate with muscle weakness [19]. The capability of US to measure tissue perfusion and echogenicity makes it a promising tool to study muscle inflammation [20]. In addition, other US domains findings such as increased fascial thickness and reduced muscle bulk have been described in IIM but are still contentious and have not been validated, largely due to lack of normal values in healthy controls [11] Similarly, published evidence based on the use of SWE suggests that muscle stiffness is reduced with increasing age and in active IIMs, but this needs replication to ensure validity [19].

This study aimed to determine if SWE and US measurements have a role in measuring inflammation or changes in diseased muscle tissue in a group of IIM patients compared to normative data for muscle groups typically affected by the disease. Firstly, this study produces normative metrics with SWE and US of selected muscles in a group of healthy adults, including the influence of age, sex, and BMI. Secondly, this study, evaluated the ability of SWE/US to detect pathology in affected muscles of patients with an established diagnosis of inclusion body myositis (IBM) or necrotising autoimmune myopathy (NAM) by comparing the IIM patients to the healthy controls. We focussed on IBM and NAM because these have been less studied by US/SWE and because they more often pose problems with confirmation of the diagnosis and treatment than other forms of IIM such as dermatomyositis and overlap syndromes.

## Methods

### Subjects

Participants (≥ 18 years of age) who were willing and able to provide written informed consent, be able to attend a clinical study visit at a single tertiary hospital centre, and attend a separate ultrasound visit at a private imaging centre. IIM patients were eligible for the study if they satisfied the 2017 European League Against Rheumatism (EULAR) classification of IIM and/or 188th Neuromuscular Centre (ENMC) diagnostic criteria for IBM and/or ENMC 2004 diagnostic criteria for immune-mediated NAM [1, 21, 22]. Forty participants, including 30 healthy controls and 10 patients with a biopsy-confirmed IIM were recruited for this study from November 2018 to March 2019. We excluded one healthy control who failed to attend the ultrasound visit. Two healthy controls were pregnant at the time of the study. Further details of the patient and control groups are provided in Supplementary Table 2. Approval for the study was granted by the South Metropolitan Health Service Human Research Ethics Committee (EC00265). All methods were performed in accordance with the relevant guidelines and regulations.

### Clinical and laboratory assessments

Data was collected on demographic and clinical characteristics, and patient-reported outcomes, including the health assessment questionnaire (HAQ) [23]; patient global assessment of disease activity visual analogue scale (patient VAS, 0–10) [24]; and physician global assessment of disease activity visual analogue scale (physician VAS, 0–10) [24]. In these three patient-reported outcome scales, higher scores indicate more severe functional impairment or disease activity. All participants underwent a full clinical examination, including manual muscle testing in 26 muscle groups (MMT26) [25]. The MMT26 is a partially validated tool to assess muscle strength at 26 sites using a 0-10-point scale, with higher scores indicating greater muscle strength (maximum score = 260). The MMT26 allows identification of muscle-specific strength scores for the deltoid, quadriceps, and the tibialis anterior. Participants also underwent routine haematological and biochemical analyses, including creatine kinase (CK) activity, and myositis autoantibody studies.

### Imaging Method/landmarks

Imaging was conducted by a sonographer who was blinded to the study groups. As previous studies have shown no significant differences in muscle stiffness (SWS) between dominant and non-dominant limbs [26], imaging of muscle landmarks with SWE, B mode US and power Doppler (PD) were performed on the participant’s right-hand side.

### Shear wave elastography

To determine muscle stiffness, SWE (recorded as SWS in m/s) was conducted using a Canon Aplio 500 machine with a linear probe set at 4 MHz frequency. Due to clinic time feasibility, SWE was only assessed in the deltoid and vastus lateralis. These two muscles were chosen because they are commonly involved in IIM [27]. SWS was recorded both at rest and with the muscle passively stretched [6]. For the deltoid, rest was defined as the position in which the elbow was flexed and rested on a pillow, and passive stretch was defined as the position in which the arm hung freely with the elbow extended [6]. In the vastus lateralis, rest was defined as the position in which the knee was extended with the patient supine on a bed [6], while passive stretch was defined as when the knee was flexed to 45 degrees (Supplementary Figs. 1 and 2).

SWS was chosen over MSM as a measure of muscle stiffness as it is devoid of assumptions that tissue density is constant at 1 g/cm ^3^ and that all tissues are isotropic and homogenous, which is not the case with muscles [26]. SWS measurement was undertaken with minimal skin pressure, and the probe held in the longitudinal plane to the muscle fibres, which is considered to be the most reliable technique [26]. The probe position for imaging the deltoid was 1/3 of the distance from the acromion to the lateral epicondyle (Supplementary Fig. 1). The vastus lateralis probe position was 1/4 of the distance from the anterior superior iliac spine (ASIS) to the superior border of the patella (Supplementary Fig. 2). In order to determine SWS, two 3mm ROI boxes were placed in a region of uniform elastogram vertical lines aiming for a standard deviation (SD) of less than 20 % of the mean speed (Supplementary Fig. 3) [17]. Care was taken not to place the ROI at the epimysium as it could affect the elasticity [26]. The final SWS value was derived from the average of the two ROIs.

### B mode ultrasound

B mode US was performed using the same Canon Aplio 500 machine with a 14 − 5 linear probe set at 14 MHz frequency. B mode US imaging was undertaken in the deltoid, flexor digitorum profundus, flexor carpi ulnaris, vastus lateralis, and the tibialis anterior. The probe was placed to capture the short axis image of each muscle, and the deltoid and vastus lateralis were landmarked as described for the SWE measurements. The probe position for the flexor digitorum profundus and flexor carpi ulnaris was 5 cm from the olecranon process (Supplementary Fig. 4). For the tibialis anterior, the probe position was on the anterior shin just proximal to the bulk of the calf (Supplementary Fig. 5).

**Fig. 1:**
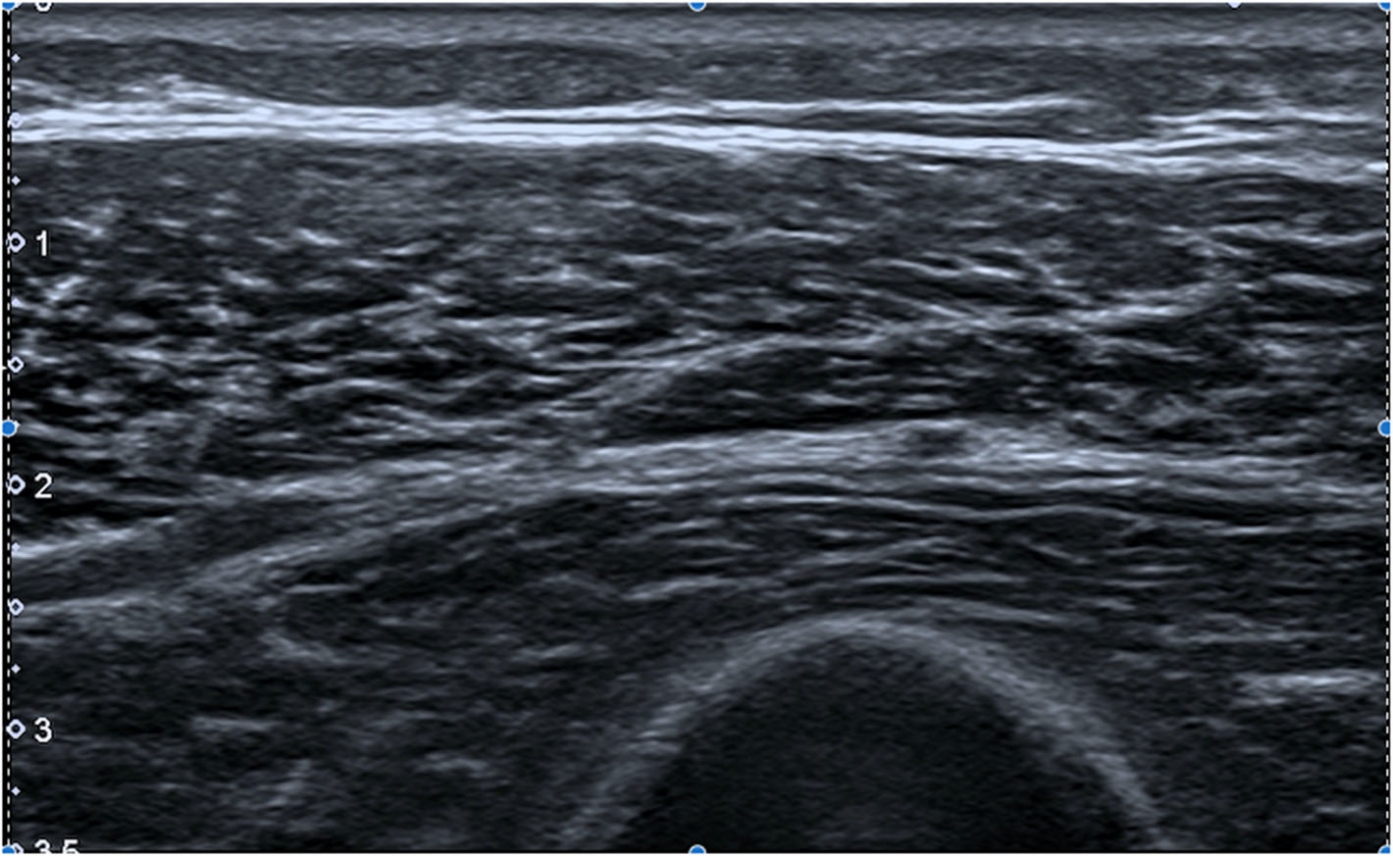
B mode Ultrasound showing a transverse view of the Vastus Lateralis in a healthy individual

**Fig. 2:**
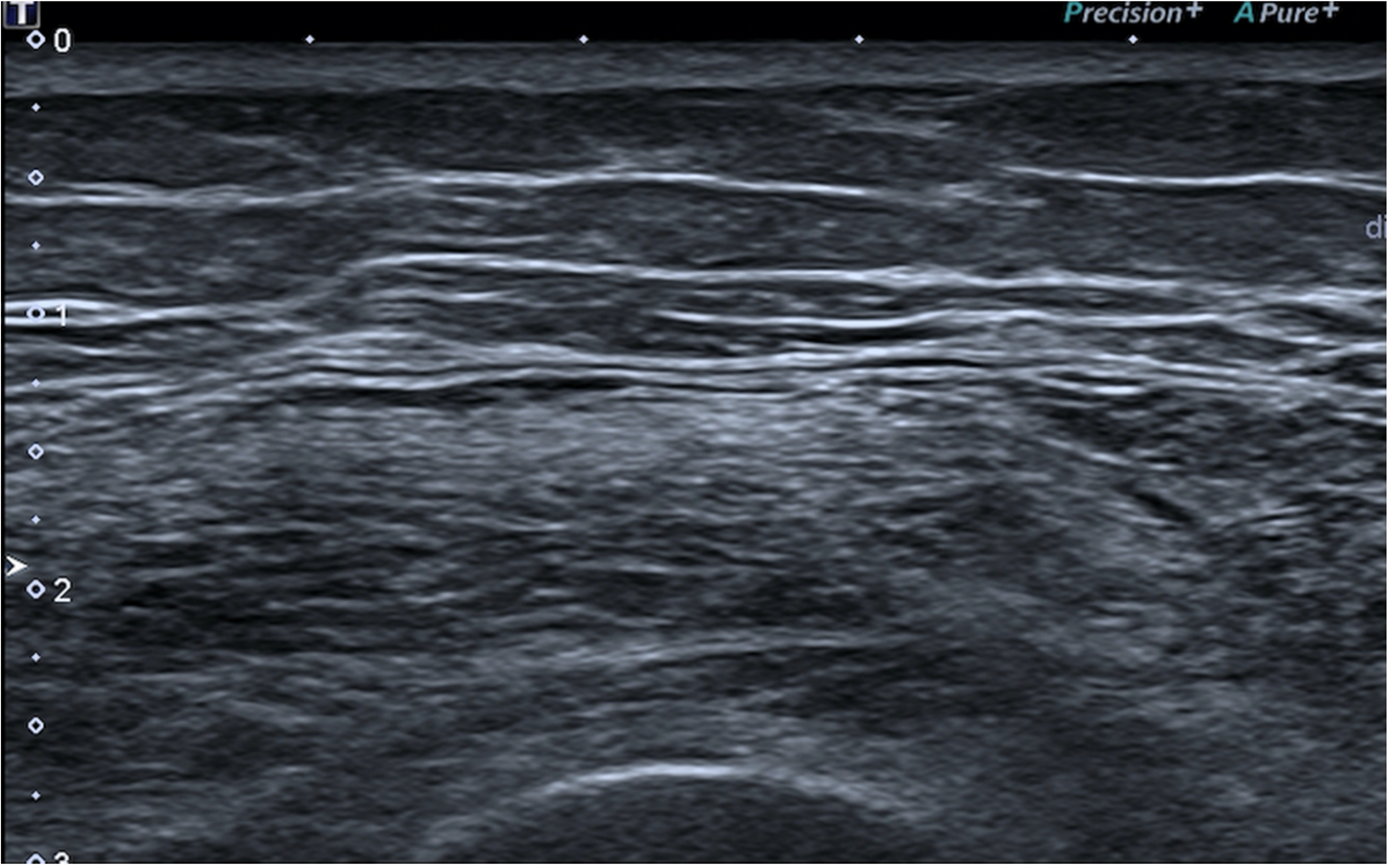
B mode Ultrasound showing a transverse view of the Vastus Lateralis in a patient with Inclusion Body Myopathy. Muscle echogenicity is increased (appears brighter) and there is loss of muscle bulk

A semi-quantitative grading of echogenicity was assigned to each muscle group using the Heckmatt visual grading score [28]. Each muscle was assigned a score of 1–4 as a representation of the quantity of echoes displayed in the greyscale image (Figs. 1 and 2) using the echogenicity of cortical bone as a visual anchor [28]. A grade of 4 indicates increased echogenicity and obliteration of cortical bone echo. Fascial thickness (aggregate of dissectible connective tissue sheaths that envelopes muscle) of each muscle was measured in millimetres using the calliper function in a homogenous area of the fascia [29]. Five samples were obtained, each at least 0.25 cm apart, and averaged [30]. Muscle bulk was measured in 3 muscles (deltoid, vastus lateralis and flexor digitorum profundus) by measuring the thickest portion of the muscle belly from the superficial fascia to the deep fascia in cross-section under the pre-determined probe positions described above [30].

### Power Doppler (PD)

The PD was assessed with a low wall filter and pulse repetition frequency (PRF) at 750 Hz. Colour gain (CG) was increased until the ROI was filled and reduced again until background noise was eliminated. PD images were obtained at rest after B mode images were taken, both in transverse and longitudinal planes. A modified semi-quantitative PD grading scale of 0–4 [31] was used, and the highest PD score was taken as the overall score for each muscle. Grade 0 was defined as no vessels seen; Grade 1: at least one intramuscular vessel seen; Grade 2 was defined as ≥5 vessels in a two-dimensional frame or a single large intramuscular vessel seen in a cross-section of > 5mm, or a segment length of > 10mm; Grade 3 = vascularity grade 2 with small clusters (≥3) of vessels; Grade 4 = appearance of frank blush, or vessel boundaries not distinguishable. Higher PD scores indicate greater degrees of vascularity.

The normal ranges for each US domain for each muscle were analysed. As age-related changes in muscle structure and function are known to commence after ≥ 50 years of age [32], the healthy control group was divided into those ≤ 50 years of age and those ≥ 50 years of age. When comparing with findings in IIM, the healthy control group was restricted to a subgroup comprising of those ≥50 years of age.

To assess the intra-observer reliability, three months after the initial assessment, a re-reading session was performed with restored images in 21 % of the cases. The analysis of the recorded images was carried out by the first author (SP) who had undergone a previous 6-month period of intensive training under the supervision of an experienced sonographer.

### Statistical methods

Continuous data are described as either median with interquartile range (IQR) or mean with confidence intervals. Continuous data were compared across IIM patients and healthy controls using the Mann-Whitney U test or One-way ANOVA. Categorical data are described as a frequency with proportion and compared across groups with the Fisher’s Exact test. Correlations were done using Spearman’s rank correlation coefficient. For statistical comparisons p values < 0.05 were considered significant. For intra-observer comparisons, the ICC was categorised as: slight agreement (0.00-0.20), fair agreement (0.21–0.40), moderate agreement (0.41–0.60), substantial agreement (0.61–0.80), and almost perfect agreement (0.81-1.00) [33]. Statistical analysis was carried out using SPSS version 24.0 (IBM Corp. in Armonk, NY).

## Results

### Normative muscle metrics in controls

Complete data for shear wave speed (muscle stiffness), echogenicity, fascial thickness, muscle bulk, and power Doppler scores (vascularity) was available for each muscle examined in 29 healthy controls (mean age 46.60 ± 16.1; female 44.80 %) (Table 1). There were no statistically significant differences between the values across the two age groups (< 50 years and ≥50 years), except that SWS in the deltoid at passive stretch was significantly lower in the ≥50 year group compared to the < 50 year group (2.80 vs. 2.47, p = 0.033). Comparison of the metrics for the individual US domains showed variation between different muscle groups. For example, SWS measure of muscle stiffness was greater in the deltoid than in the vastus lateralis and was higher in the stretched than in the rest state. Echogenicity and power Doppler values varied over a broad range (1–3 and 0–4 respectively) in the muscles examined. In this study, echogenicity of > 1 is likely outside of the normal range in all muscles except the vastus lateralis where the threshold was > 2. Fascial thickness was greater in the proximal limb muscles (deltoid, vastus lateralis) than in the distal muscles (flexor digitorum profundus, flexor carpi ulnaris, tibialis anterior).

**Table 1:** Descriptive data in the healthy control group showing normal ranges for each ultrasound domain in each muscle examined, presented as median, interquartile range, minimum and maximum values

	Median (IQR), Min-Max		p- value^a^ (^comparing medians)^
	**Age < 50**	**Age ≥50**	
**Fascial Thickness (mm)**			
Deltoid	0.76(0.66–0.84)0.50–0.92	0.72(0.64–0.76)0.48–0.90	0.123
Vastus Lateralis	0.80(0.68–0.92)0.64 -1.00	0.86(0.70–0.93)0.50-1.00	0.627
Tibialis Anterior	0.59(0.50–0.70)0.48–0.72	0.61(0.56–0.71)0.52–0.72	0.477
Flexor Digitorum Profundus	0.54(0.48–0.63)0.42–0.70	0.59(0.52–0.64)0.46–0.68	0.329
Flexor Carpi Ulnaris	0.60(0.56–0.64)0.36–0.74	0.55(0.48–0.72)0.37–0.80	0.451
**Muscle Bulk (mm)**			
Deltoid	19.80(14.40–22.20)9.40–27.3	17.30(15.88–19.35)11.4–27.1	0.354
Vastus Lateralis	20.15(17.20–2150)13.2–25.3	17.60(14.28–22.65)13.1–24.4	0.377
Flexor Digitorum Profundus	10.55(9.20–11.30)8.30–15.2	12.60(9.28–14.90)8.00-17.1	0.252
**Shear Wave Speed (m/s)**			
Deltoid (rest)	2.31(1.94–2.55)1.58–2.83	2.14(1.79–2.47)1.36–2.85	0.652
Deltoid (Stretch)	2.80(2.37–3.24)1.65–4.68	2.47(2.18–2.63)1.72–2.97	0.333
Vastus Lateralis (Rest)	1.70(1.63–1.75)1.59–3.34	1.74(1.57–1.97)1.34–2.08	0.747
Vastus Lateralis (Stretch)	1.79(1.70–1.97)1.47–3.08	1.90(1.81–2.04)1.62–2.43	0.270
**Echogenicity (1–4)**			P value^b^ (comparing medians)
Deltoid	1(1,1)1–3	1 (1,1)1–3	0.553
Vastus Lateralis	1(1, 1)1–2	1(1–2)1–3	0.451
Flexor digitorum profundus	1(1,1) 1–1	1 (1,1) 1–2	0.533
Flexor carpi ulnaris	1(1–1)1–1	1 (1,1) 1-1	0.747
Tibialis anterior	1(1–1)1–2	1 (1,2)1–2	0.331
**Power Doppler (0–4)**			P value ^b^ (comparing medians)
Deltoid	0 (0–1) 0–2	0 (0,0) 0–2	0.134
Vastus Lateralis	1 (0,1) 0–1	1(0–1) 0–2	0.949
Flexor Digitorum Profundus	0 (0,1) 0–2	1(0–1) 0–2	0.477
Flexor Carpi Ulnaris	1 (0,1) 0–3	1(0–1) 0–4	0.533
Tibialis Anterior	1 (0,1) 0–1	1(0–1) 0–2	0.252

*IQR *interquartile range, min-max: minimum-maximum, *P* value^a^: Mann-Whitney test, *P* value^b^: Fisher Exact Test

### Influence of age, sex and BMI on normative muscle metrics

The influence of age, sex and BMI on healthy muscle is presented in Supplementary Table 1. Age was found to have significant correlations with a number of US domains. SWS in the deltoid at passive stretch correlated inversely with age (Rs: -0.40, P = 0.030). Echogenicity in the flexor digitorum profundus was positively correlated with age (Rs. 0.38, P = 0.045). Similarly, PD was positively correlated with age in the deltoid (P = 0.032). Females had lower muscle bulk in the deltoid (P = 0.022). No gender influences were noted for SWS, echogenicity or PD. Higher BMI was associated with lower SWS (Rs. -0.45, P = 0.018) and higher echogenicity (Rs. 0.69, P < 0.001) in the deltoid, but was not associated with PD score in any muscle group (p > 0.10).

### Comparison of muscle metrics in IBM/NAM cases and healthy controls ≥ 50 years

Muscle imaging findings were compared in 13 healthy controls ≥ 50 years (mean age 61.00 ± 7.47) and 10 participants with IIM (mean age 67.00+/-8.18 years); 5 with IBM and 5 with NAM). There were more males in the IIM group (n = 8/10, 80.0 %) and more females in the healthy control ≥ 50 years group (n = 8/14, 57.1 %), which was not statistically significant (P = 0.069). Participants with IIM recorded a lower MMT26 (i.e., weaker muscles), a higher participant and physician global activity VAS (i.e., more disease activity), HAQ scores (i.e., more disability), and a higher average serum CK level, these differences all being statistically significant (p < 0.002). IBM patients had a lower BMI and CK levels compared to NAM, and higher participant and physician VAS and higher HAQ scores, which were all statistically significant (p < 0.049) (Supplementary Table 2).

In-group comparisons for the deltoid muscle between the five NAM cases and healthy controls ≥ 50 years, no significant differences were apparent in SWS (P = 0.099), fascial thickness (P = 0.220), muscle bulk (P = 0.727), echogenicity (P = 0.199) or PD score (P = 0.086) (Supplementary Table 3). However, fascial thickness was significantly reduced in the tibialis anterior (P = 0.002) and flexor digitorum profundus (P = 0.004). Analysis of SWS values on a case-to-case basis showed that values in all five cases were lower than the mean for the and healthy controls ≥ 50 years group, particularly in the stretched muscle condition (Table 2), and SWS values were also lower in the vastus lateralis in a number of cases (Table 2).

**Table 2:** Case-by-case analysis of shear wave speed (SWS) in the IBM and NAM groups with corresponding age and muscle weakness

Study Subtype	Age	SWSD Rest-m/s	SWSD Stretch-m/s	SWSVL Rest-m/s	SWSVL Stretch-m/s	MMTD /10	MMT quads/10
NAM	69	1.75	2.04	1.95	2.04	9	10
NAM	71	1.75	1.79	1.89	2.87	9	8
NAM	52	1.77	2.03	1.68	1.70	6	7
NAM	56	2.67	2.29	1.67	1.81	10	8
NAM	70	2.17	2.12	1.55	2.28	7	8
IBM	76	1.78	4.05	1.83	4.19	7	5
IBM	77	2.56	2.10	1.50	1.77	10	9
IBM	68	1.61	2.51	1.41	3.43	10	7
IBM	61	1.81	2.25	1.71	2.30	5	9
IBM	70	1.66	2.59	2.16	1.30	10	7
HC Mean+/-SD	61.00, 7.47	2.14+/-0.43	2.40+/-0.36	1.74+/-0.24	1.93+/-0.20	10	10

*D *deltoid, *VL *vastus lateralis, *quads *quadriceps femoris, *SWS *shear wave speed, *m/s *metres per second, *MMT *manual muscle testing (maximum score 10), *NAM * necrotising autoimmune myopathy, *IBM *inclusion body myositis, *HC *healthy controls, *SD *standard deviation

In IBM, the findings in the flexor digitorum profundus, tibialis anterior and vastus lateralis muscles were compared with controls, based on the known predilection of IBM for these muscles. In the flexor digitorum profundus, echogenicity was increased (P = 0.022) and fascial thickness was reduced (P = 0.04), while PD scores (P = 0.881) did not differ significantly between the two groups. In the tibialis anterior, echogenicity was increased (P = 0.004), fascial thickness was significantly reduced (P = 0.002), while PD scores did not differ between groups (P = 0.518). In the vastus lateralis muscle bulk was significantly reduced (P = 0.033). Mean SWS values in the vastus lateralis and deltoid did not differ significantly from the healthy controls ≥ 50 years group (supplementary Table 3), but case-to-case analysis showed that in a number of cases, SWS values were lower than the mean for the healthy controls ≥ 50 years group in the resting state, and higher in the stretched state (Table 2).

### Intra-observer reliability

The images of seven healthy controls and one patient with IIM were reviewed for reliability. Intra-observer variability for scoring echogenicity on B mode ultrasound demonstrated perfect intraclass correlation coefficient agreement (kappa 1.00) [33] in all of the deltoid, vastus lateralis, flexor digitorum profundus, flexor carpi ulnaris and tibialis anterior muscles. PD scoring showed substantial agreement in vastus lateralis (kappa 0.85), flexor digitorum profundus (kappa 0.87), deltoid (kappa 0.67) and moderate agreement in the flexor carpi ulnaris (kappa 0.43).

## Discussion

### Normative data

Although SWE, B mode US and PD have been studied previously in patients with musculoskeletal disorders (6, 26, 29, 30), to our knowledge, this is the first study in which they have been used in combination in a group of healthy controls to establish normal metrics and US profiles for selected upper and lower limb muscles which are typically affected in IIM. Previous studies of SWE and ultrasound in immune-mediated IIMs have focussed on dermatomyositis, polymyositis and overlap syndromes, and there is a paucity of studies of IBM and the more recently characterised subtype of NAM. The primary objective was to evaluate the properties of healthy muscle with SWE, B mode US and PD, and to undertake an exploratory assessment of how they are altered in a small group of patients with IBM and NAM which have not been studied previously. Normative data in the adult population are lacking and most previous studies have focussed on the paediatric population which may have different muscle metrics. In our study, healthy controls generally showed low grades of echogenicity and PD in all muscles examined with an age influence of echogenicity in the flexor digitorum profundus and power Doppler at the deltoid.). We found that echogenicity > 1 is likely to be of significance in all muscles studied, except the vastus lateralis which had a higher threshold of > 2. The increased threshold in the quadriceps may be due to the accelerated sarcopenic effects in the quadriceps muscles and increasing myosteatosis compared to its counterparts [19, 34]. A PD score > 1 was also likely significant in all muscles, but it is unclear whether this is necessarily pathological as there may be other influences on muscle vascularity such as exercise, temperature, medications and hyperdynamic states that need further study.

### Influences of age, sex and BMI on healthy muscle

Previous published data have indicated that age may be an influence on muscle ultrasound properties [35, 36], especially in men [34]. Therefore, we examined the impact of age on healthy controls in this study. Our SWE data in healthy controls indicated an age-associated decline in deltoid SWS at passive stretch but not at rest, indicating a reduction in muscle stiffness. As muscles at rest and stretch may have different physical properties [37], SWS should therefore be considered contextually with regards to whether the muscle is stretched or not [37] [15]. The decline in SWS in the deltoid with age in healthy controls has been reported by Alfuraih et al. but in contrast to our findings, the age-associated decline was seen at rest and not in the passively stretched state [19]. We found no age-related changes in SWE stiffness in the vastus lateralis, in contrast to the study by Alfuraih et al. which showed a decline in stiffness in the vastus lateralis at rest with age. This difference may be related to differences in sample size, age distributions, and different limb positions during the procedure and definitions of stretch and rest.

In our study, BMI was found to influence muscle stiffness and echogenicity in the deltoid. Higher echogenicity and lower muscle stiffness were associated with a higher BMI in the deltoid. The association of higher BMI and higher echogenicity is expected [38] but the predilection to the deltoid is interesting and warrants further study. Apart from the expected greater muscle bulk in the deltoid in males, there were no gender-related differences in muscle stiffness or other US measures in other muscles.

### Comparison between healthy controls ≥ 50 years and IIM muscles

In this preliminary work considering healthy controls ≥ 50 years, compared to IIM participants, differential changes in SWE muscle stiffness were found in different muscles in patients with NAM and IBM. In NAM, SWS was reduced relative to controls in both the stretched deltoid and vastus lateralis, which are both proximal limb muscles affected by the disease, but the group differences were not statistically significant, most likely because of the small patient numbers. In IBM, the group changes in SWS in the vastus lateralis, which is one of the muscles most severely affected by the disease [39] were not significantly different from healthy controls ≥ 50 years. However, there was a greater variability in SWS than in controls in both the resting and stretched muscle conditions. Our preliminary findings suggest that there are changes in the physical properties of affected muscles in these forms of IIM, but these require further investigation in larger patient cohorts in which correlations with parameters of disease severity and duration can be investigated. SWE findings of reduced muscle stiffness in IIM compared to healthy controls has been demonstrated in a previous study, by Alfuraih et al.[6, 19], but the study did not include patients with NAM or IBM.

Other changes in the muscle properties in the IIM group included increased echogenicity, which was present in almost all muscle groups examined compared to healthy controls ≥ 50 years. In the acute setting, increased echogenicity may be the result of inflammation or fatty replacement (myosteatosis) [11, 40]. However, the people with IIM in this study were all prevalent cases, without clinical evidence of inflammation, and therefore the increased echogenicity in these chronically affected muscles is likely to be a result of myosteatosis in which there is progressive loss of muscle fibres, as is known to occur in IBM [11, 41]. It is thought that fatty replacement increases echogenicity as opposed to intramuscular fibrosis [42]. However, the US correlation with interstitial muscle fibrosis has not been clearly defined and further studies that involve comparison with pathology would be needed to evaluate this.

Another finding of note was a reduction in fascial thickness in the flexor digitorum profundus and tibialis anterior muscles in IIM compared to healthy controls. To date, published data regarding fascial thickness has focused on the IIM subsets of DM and PM, and has described an increase in fascial thickness [43], which is in contrast to our findings in NAM and IBM. We are unaware of any previous published data on fascial thickness in NAM and IBM and the factors responsible for a reduction in fascial thickness are unclear. The increased fascial thickness in DM is thought to occur as a result of vascular endothelial growth factor (VEGF) expressing cells and angiogenesis in the fascia (37). However, given VEGF is not a prominent cytokine in IBM and NAM, it is intuitive that fascial thickness would not be increased in IBM and NAM [44]. The reason for the facial thinning is not clear; the role of prednisolone and the underlying disease pathology on fascia in these subtypes is unknown and may have an influence on the fascia.

No differences in PD signal were seen between healthy controls and people with IIM. A single previous published study reported increased PD signal in acute IIM [45]. A few possibilities could explain this discordance. The healthy control group in the present study reported higher exercise intensity on history (compared to IIM), which could influence muscle vascularity. Exercise has been reported to increase intramuscular vascularity as perfusion increases [31]. Additionally, there were two patients in our study who were pregnant. We are unable to find published data to demonstrate that pregnancy, a hyperdynamic state, definitively increases vascularity in muscle, but it remains a possibility. The chosen PD grading score, which includes the presence of large intramuscular vessels, (as opposed to just fine tissue vascularity) may also have been a factor. Finally, in contrast to the published study (37) which examined acute, incident IIM (although exclusive of NAM), our cohort was a prevalent group so that muscle inflammation may have been reduced as a result of treatment.

### Limitations

This study has a number of limitations. Firstly, as SWE and US are operator-dependent, they can be affected by subjective changes in the degree of pressure applied to the muscle being examined. However, care was taken to minimise this by using a standardised predefined and previously validated protocol for each muscle examined. Secondly, the study included only patients with NAM and IBM and did not include other forms of IIM such as dermatomyositis, polymyositis or overlap syndromes. Thirdly, as an exploratory pilot study, the number of subjects included in the IIM group was small (n = 10), and the study was not sufficiently powered for subgroup analysis or correlations with effects of treatment or with parameters of disease activity or clinical outcome measures. Overall, while a high degree of inter-observer reliability and high ICCs was demonstrated in the analysis of ultrasound images, the possibility that the retrospective analysis of stored images may have affected the ICCs for the power doppler analysis cannot be excluded.

## Conclusions

This study provides normative SWE and US data for healthy controls at sites clinically relevant to IBM and NAM. We have shown influence of age and BMI on SWE and US properties in the deltoid which is an important consideration when choosing a muscle to study. Future work investigating whether ultrasound has the potential to be a clinically useful tool should examine its performance when compared to current diagnostic and clinical outcome measures with particular attention to construct validity, longitudinal discrimination and feasibility.

## Supplementary Information

**Additional file 1: Supplementary Table 1**: Age, gender and BMI influences in healthy controls.

**Additional file 2: Supplementary Table 2**: Baseline characteristics of IIM patients and subgroup of healthy controls over 50 years of age.

**Additional file 3: Supplementary Table 3**: Discrimination of ultrasound domains between IIM and healthy controls.

**Additional file 4: Supplementary Figure 1**: The Deltoid probe position was 1/3 of the distance from the acromion to the lateral epicondyle.

**Additional file 5: Supplementary Figure 2**: The Vastus Lateralis probe position was ¼ of the distance from the anterior superior iliac spine (ASIS) to the superior border of the patella.

**Additional file 6: Supplementary Figure 3**: Shear wave speed in the deltoid of a 28-year-old healthy female at rest showing the colour blue, which indicates lower muscle stiffness.

**Additional file 7: Supplementary Figure 4**: The Flexor Digitorium Profundus and Flexor Carpi Ulnaris probe position was 5 cm from olecranon process.

**Additional file 8: Supplementary Figure 5**: The Tibialis Anterior muscle probe position is anterior shin just proximal to the calf bulk.

## Data Availability

All data generated or analysed during this study are included in this published article [and its supplementary information files.

## Abbreviations

IIM: Aidiopathic inflammatory myopathies
SWE: Shear wave elastography
MSM: Muscle shear modulus
SWS: Shear wave speed
PD: Power Doppler
DM: Dermatomyositis
PM: Polymyositis
IBM: Inclusion body myositis
NAM: Necrotising autoimmune myositis
D: Deltoid
FDP: Flexor digitorum profundus
FCU: Flexor carpi ulnaris
VL: Vastus lateralis
TA: Tibialis anterior
BMI: Body mass index
MMT26: Manual muscle testing for 26 muscle
VAS: Visual analogue scale
HAQ: Health assessment questionnaire
