# Supplementary material for: Muscle shear wave elastography, conventional B mode and power doppler ultrasonography in healthy adults and patients with autoimmune inflammatory myopathies: a pilot cross-sectional study
Source: BMC Musculoskelet Disord. 2021 Jun 12;22:537. doi: 10.1186/s12891-021-04424-0 (PMC8199828; doi:10.1186/s12891-021-04424-0)
Supplement: Supplementary file 1 — Additional file 1: Supplementary Table 1: Age, gender and BMI influences in healthy controls. [file 12891_2021_4424_MOESM1_ESM.docx]

***Supplementary Table 1*:** Age, gender and BMI influences in healthy controls

|  | | Overall (n=29) | Correlation with age  (Spearman’s correlation) | | Gender | | | Correlation with BMI  (spearman’s correlation) | |
| --- | --- | --- | --- | --- | --- | --- | --- | --- | --- |
|  |  |  | Rs | p-value | Males  Mean+/-SD | Females  Mean +/-SD | p-value^a^ | Rs | p-  value |
| **Shear wave speed (SWS) m/s** | |  |  |  |  |  |  |  |  |
| Deltoid (rest) | | 2.20 ± 0.41 | -0.14 | 0.460 | 2.21 ± 0.39 | 2.20 ± 0.45 | 0.940 | -0.04 | 0.084 |
| Deltoid (stretch) | | 2.67 ± 0.67 | -0.40 | **0.030** | 2.88 ± 0.72 | 2.41 ± 0.51 | 0.059 | -0.44 | **0.018** |
| Vastus Lateralis (rest) | | 1.77 ± 0.35 | 0.03 | 0.870 | 1.83 ± 0.43 | 1.68 ± 0.21 | 0.266 | 0.12 | 0.556 |
| Vastus Lateralis (stretch) | | 1.93 ± 0.34 | 0.28 | 0.140 | 2.00 ± 0.41 | 1.85 ± 0.24 | 0.256 | 0.42 | 0.026 |
| **Muscle Bulk (mm)** | |  |  |  |  |  |  |  |  |
| Deltoid | | 0.72 ± 0.11 | -0.32 | 0.090 | 0.72 ± 0.14 | 0.73 ± 0.07 | 0.888 | 0.38 | **0.043** |
| Vastus Lateralis | | 0.80 ± 0.14 | 0.05 | 0.790 | 0.80 ± 0.13 | 0.80 ± 0.17 | 0.969 | 0.03 | 0.088 |
| Tibialis Anterior | | 0.61 ± 0.08 | -0.03 | 0.860 | 0.61 ± 0.09 | 0.62 ± 0.07 | 0.626 | 0.13 | 0.496 |
| Flexor Digitorum Profundus | | 0.56 ± 0.08 | 0.14 | 0.460 | 0.57 ± 0.08 | 0.55 ± 0.07 | 0.564 | 0.04 | 0.086 |
| Flexor Carpi Ulnaris | | 0.58 ± 0.11 | -0.06 | 0.760 | 0.60 ± 0.10 | 0.56 ± 0.12 | 0.406 | -0.16 | 0.428 |
| **Fascial thickness (mm)** | |  |  |  |  |  |  |  |  |
| Deltoid | | 18.18 ± 4.60 | -0.28 | 0.140 | 20.31 ± 4.84 | 16.44 ± 3.70 | **0.022** | 0.25 | 0.209 |
| Vastus Lateralis | | 18.65 ± 3.65 | -0.29 | 0.130 | 18.67 ± 3.30 | 18.63 ± 4.19 | 0.978 | 0.12 | 0.532 |
| Flexor Digitorum Profundus | | 11.42 ± 2.45 | 0.17 | 0.370 | 10.83 ± 2.44 | 12.15 ± 2.35 | 0.149 | -0.04 | 0.080 |
| **Echogenicity (1-4)** | |  |  |  |  |  |  |  |  |
| Deltoid | Normal | 25 (86.20%) | 0.25 | 0.195 | 13 (81.30%) | 12 (92.30%) | 0.417 | 0.69 | **0.000** |
|  | Mild | 2 (6.90%) |  |  | 1 (6.30%) | 1 (7.70%) |  |  |  |
|  | Moderate | 2 (6.90%) |  |  | 2 (12.50%) | 0 (0.00%) |  |  |  |
|  | High | 0 (0.00%) |  |  | 0 (0.00%) | 0 (0.00%) |  |  |  |
| Vastus Lateralis | Normal | 21 (72.40%) | 0.35 | 0.065 | 11 (68.8%) | 10 (76.90%) | 0.641 | 0.14 | 0.486 |
|  | Mild | 7 (24.10%) |  |  | 4 (25.0%) | 3 (23.10%) |  |  |  |
|  | Moderate | 1 (3.40%) |  |  | 1 (6.3%) | 0 (0.00%) |  |  |  |
|  | High | 0 (0.00%) |  |  | 0 (0.0%) | 0 (0.00%) |  |  |  |
| Flexor Digitorum Profundus | Normal | 27 (93.10%) | 0.38 | **0.045** | 15 (93.8%) | 12 (92.30%) | 0.879 | 0.31 | 0.220 |
|  | Mild | 2 (6.90%) |  |  | 1 (6.3%) | 1 (7.70%) |  |  |  |
|  | Moderate | 0 (0.00%) |  |  | 0 (0.0%) | 0 (0.00%) |  |  |  |
|  | High | 0 (0.00%) |  |  | 0 (0.0%) | 0 (0.00%) |  |  |  |
| Flexor Carpi Ulnaris | Normal | 28 (96.60%) | 0.21 | 0.263 | 15 (93.8%) | 13 (100.00%) | 0.359 | -0.08 | 0.710 |
|  | Mild | 1 (3.40%) |  |  | 1 (6.3%) | 0 (0.00%) |  |  |  |
|  | Moderate | 0 (0.00%) |  |  | 0 (0.0%) | 0 (0.00%) |  |  |  |
|  | High | 0 (0.00%) |  |  | 0 (0.0%) | 0 (0.00%) |  |  |  |
| Tibialis Anterior | Normal | 24 (82.80%) | 0.32 | 0.088 | 14 (87.5%) | 10 (76.90%) | 0.453 | 0.14 | 0.487 |
|  | Mild | 5 (17.20%) |  |  | 2 (12.5%) | 3 (23.10%) |  |  |  |
|  | Moderate | 0 (0.00%) |  |  | 0 (0.0%) | 0 (0.00%) |  |  |  |
|  | High | 0 (0.00%) |  |  | 0 (0.0%) | 0 (0.00%) |  |  |  |
| **Power Doppler (0-4)** |  |  |  |  |  |  |  |  |  |
| Deltoid | Normal | 20 (69.00%) | -0.40 | **0.032** | 10 (62.5%) | 10 (76.90%) | 0.548 | -0.06 | 0.769 |
|  | Mild | 8 (27.60%) |  |  | 5 (31.3%) | 3 (23.10%) |  |  |  |
|  | Moderate | 1 (3.40%) |  |  | 1 (6.3%) | 0 (0.00%) |  |  |  |
|  | High | 0 (0.00%) |  |  | 0 (0.0%) | 0 (0.00%) |  |  |  |
|  | Highest | 0 (0.00%) |  |  | 0 (0.0%) | 0 (0.00%) |  |  |  |
| Vastus Lateralis | Normal | 12 (41.40%) | 0.00 | 0.998 | 8 (50.0%) | 4 (30.80%) | 0.360 | -0.27 | 0.172 |
|  | Mild | 16 (55.20%) |  |  | 8 (50.0%) | 8 (61.50%) |  |  |  |
|  | Moderate | 1 (3.40%) |  |  | 0 (0.0%) | 1 (7.70%) |  |  |  |
|  | High | 0 (0.00%) |  |  | 0 (0.0%) | 0 (0.00%) |  |  |  |
|  | Highest | 0 (0.00%) |  |  | 0 (0.0%) | 0 (0.00%) |  |  |  |
| Flexor Digitorum Profundus | Normal | 17 (58.60%) | 0.11 | 0.587 | 10 (62.5%) | 7 (53.80%) | 0.895 | 0.06 | 0.767 |
|  | Mild | 10 (34.50%) |  |  | 5 (31.3%) | 5 (38.50%) |  |  |  |
|  | Moderate | 2 (6.90%) |  |  | 1 (6.3%) | 1 (7.70%) |  |  |  |
|  | High | 0 (0.00%) |  |  | 0 (0.0%) | 0 (0.00%) |  |  |  |
|  | Highest | 0 (0.00%) |  |  | 0 (0.0%) | 0 (0.00%) |  |  |  |
| Flexor Carpi Ulnaris | Normal | 12 (41.40%) | 0.3500 | 0.062 | 8 (50.0%) | 4 (30.80%) | 0.360 | 0.17 | 0.381 |
|  | Mild | 14 (48.30%) |  |  | 8 (50.0%) | 6 (46.20%) |  |  |  |
|  | Moderate | 1 (3.40%) |  |  | 0 (0.0%) | 1 (7.70%) |  |  |  |
|  | High | 1 (3.40%) |  |  | 0 (0.0%) | 1 (7.70%) |  |  |  |
|  | Highest | 1 (3.40%) |  |  | 0 (0.0%) | 1 (7.70%) |  |  |  |
| Tibialis Anterior | Normal | 11 (37.90%) | 0.02 | 0.908 | 3 (18.8%) | 8 (61.50%) | 0.057 | -0.18 | 0.353 |
|  | Mild | 13 (44.80%) |  |  | 9 (56.3%) | 4 (30.80%) |  |  |  |
|  | Moderate | 5 (17.20%) |  |  | 4 (25.0%) | 1 (7.70%) |  |  |  |
|  | High | 0 (0.00%) |  |  | 0 (0.0%) | 0 (0.00%) |  |  |  |
|  | Highest | 0 (0.00%) |  |  | 0 (0.0%) | 0 (0.00%) |  |  |  |

Statistically significant: P value (p) <0.05, ^a^: P-value for one way ANOVA, SD: standard deviation
