# Supplementary material for: Muscle shear wave elastography, conventional B mode and power doppler ultrasonography in healthy adults and patients with autoimmune inflammatory myopathies: a pilot cross-sectional study
Source: BMC Musculoskelet Disord. 2021 Jun 12;22:537. doi: 10.1186/s12891-021-04424-0 (PMC8199828; doi:10.1186/s12891-021-04424-0)
Supplement: Supplementary file 2 — Additional file 2: Supplementary Table 2: Baseline characteristics of IIM patients and subgroup of healthy controls over 50 years of age. [file 12891_2021_4424_MOESM2_ESM.docx]

***Supplementary table 2:*** Baseline characteristics of IIM patients and subgroup of healthy controls over 50 years of age

|  | **IIM (n=10)** | **Healthy controls (n=13)** | **p-value ^a^** | **IBM (n=5)** | **NAM (n=5)** | **p-value ^b^** |
| --- | --- | --- | --- | --- | --- | --- |
| **Age, mean (SD)** | 67.00 (8.18) | 61.00 (7.47) | 0.076 | 70.40 (6.50) | 63.60 (8.91) | 0.082 |
| **Male, n (%)** | 8 (80.00%) | 6 (42.90%) | 0.069 | 5 (100.00%) | 3 (60.00%) | 0.084 |
| **Female, n (%)** | 2 (20.00%) | 8 (57.10%) |  | 0 (0.0%) | 2 (40.00%) |  |
| **Body Mass Index, mean (SD)** | 27.80 (6.19) | 26.83 (3.74) | 0.644 | 24.20 (3.25) | 31.40 (6.57) | **0.049** |
| **Manual Muscle Testing 26, median (IQR)** | 203.00(181.00, 223.00) | 260.00 (260.00, 260.00) | **<0.001** | 203.00 (175.00, 221.50) | 200.50 (181.00, 238.50) | **0.001** |
| **Participant VAS (10cm), median (IQR)** | 4.33 (1.50, 6.55) | 0.00 (0.00, 0.20) | **0.002** | 5.75 (5.00, 7.80) | 1.50 (1.10, 3.65) | **0.004** |
| **Physician VAS (10cm), median (IQR)** | 3.08 (1.90, 4.40) | 0.00 (0.00, 0.00) | **<0.001** | 3.80 (2.35, 3.90) | 1.90 (0.45, 4.40) | **<0.001** |
| **HAQ, median (IQR)** | 0.53 (0.30, 0.85) | 0.00 (0.00, 0.00) | **0.001** | 0.85 (0.30, 1.15) | 0.50 (0.45, 0.55) | **0.003** |
| **Creatine Kinase, median (IQR)** | 278.5 (221.0, 696.0) | 127.5 (63.0, 137.0) | **0.002** | 221.0 (201.0, 258.0) | 636.0 (299.0, 725.0) | **0.005** |
| ^a^ One-way ANOVA, Mann-Whitney U, Fisher's Exact test; ^b^ One-way ANOVA, Kruskal-Wallis, Fishers' Exact test | | | | | | |

IIM: idiopathic inflammatory myopathy, IBM: inclusion body myositis, NAM: necrotising autoimmune myopathy, statistically significant p<0.05, IQR: inter quartile range
