# Supplementary material for: Muscle shear wave elastography, conventional B mode and power doppler ultrasonography in healthy adults and patients with autoimmune inflammatory myopathies: a pilot cross-sectional study
Source: BMC Musculoskelet Disord. 2021 Jun 12;22:537. doi: 10.1186/s12891-021-04424-0 (PMC8199828; doi:10.1186/s12891-021-04424-0)
Supplement: Supplementary file 3 — Additional file 3: Supplementary Table 3: Discrimination of ultrasound domains between IIM and healthy controls. [file 12891_2021_4424_MOESM3_ESM.docx]

***Supplementary Table 3*:** Discrimination of ultrasound domains between IIM and healthy controls

| **Ultrasound domains** |  | **HC (n=13)**  **(Mean +/-SD)** | **IIM (n=10)**  **(Mean+/-SD)** | **p-value** | **IBM (n=5)**  **(Mean+/-SD)** | **NAM (n=5)**  **(Mean+/-SD)** | **p-value** |
| --- | --- | --- | --- | --- | --- | --- | --- |
| **SWS Deltoid rest (m/s)** |  | 2.14 ± 0.43 | 1.95 ± 0.38 | 0.294 | 1.88 ± 0.39 | 2.02 ± 0.40 | 0.510 |
| **SWS Deltoid stretch (m/s)** |  | 2.40 ± 0.36 | 2.38 ± 0.63 | 0.925 | 2.70 ± 0.78 | 2.05 ± 0.18 | 0.099 |
| **SWS Vastus Lateralis rest (m/s)** |  | 1.74 ± 0.24 | 1.74 ± 0.23 | 0.930 | 1.72 ± 0.30 | 1.75 ± 0.17 | 0.982 |
| **SWS Vastus Lateralis stretch (m/s)** |  | 1.93 ± 0.20 | 2.37 ± 0.89 | 0.081 | 2.60 ± 1.19 | 2.14 ± 0.47 | 0.107 |
| **Fascial thickness Deltoid mean (mm)** |  | 0.70 ± 0.11 | 0.61 ± 0.17 | 0.139 | 0.65 ± 0.15 | 0.57 ± 0.19 | 0.220 |
| **Fascial thickness Vastus Lateralis mean (mm)** |  | 0.81 ± 0.16 | 0.81 ± 0.16 | 0.970 | 0.85 ± 0.20 | 0.76 ± 0.13 | 0.720 |
| **Fascial thickness Tibialis Anterior mean (mm)** |  | 0.63 ± 0.07 | 0.53 ± 0.11 | **0.012** | 0.46 ± 0.11 | 0.59 ± 0.05 | **0.002** |
| **Fascial thickness Flexor Digitorum Profundus mean (mm)** |  | 0.58 ± 0.07 | 0.45 ± 0.09 | **0.001** | 0.46 ± 0.12 | 0.44 ± 0.06 | **0.004** |
| **Fascial Thickness Flexor Carpi Ulnaris mean** |  | 0.58 ± 0.14 | 0.50 ± 0.07 | 0.115 | 0.49 ± 0.07 | 0.50 ± 0.09 | 0.293 |
| **Muscle bulk Deltoid (mm)** |  | 17.54 ± 3.88 | 16.60 ± 2.73 | 0.520 | 16.06 ± 2.19 | 17.14 ± 3.34 | 0.727 |
| **Muscle bulk Vastus Lateralis (mm)** |  | 18.01 ± 4.09 | 15.01 ± 4.25 | 0.095 | 12.42 ± 3.23 | 17.60 ± 3.67 | **0.033** |
| **Muscle bulk Flexor Digitorum Profundus (mm)** |  | 12.17 ± 2.87  n (%) | 13.10 ± 3.14  n (%) | 0.460 | 12.30 ± 2.39  n (%) | 13.90 ± 3.85  n (%) | 0.540 |
| **Echogenicity Deltoid** | 1 | 11 (78.60%) | 5 (50.00%) | 0.337 | 3 (60.00%) | 2 (40.00%) | 0.199 |
|  | 2 | 2 (14.30%) | 3 (30.00%) |  | 2 (40.00%) | 1 (20.00%) |  |
|  | 3 | 1 (7.10%) | 2 (20.00%) |  | 0 (0.00%) | 2 (40.00%) |  |
|  | 4 | 0 (0.00%) | 0 (0.00%) |  | 0 (0.00%) | 0 (0.00%) |  |
| **Echogenicity Vastus Lateralis** | 1 | 9 (64.30%) | 2 (20.00%) | 0.056 | 1 (20.00%) | 1 (20.00%) | 0.219 |
|  | 2 | 4 (28.60%) | 4 (40.00%) |  | 2 (40.00%) | 2 (40.00%) |  |
|  | 3 | 1 (7.10%) | 4 (40.00%) |  | 2 (40.00%) | 2 (40.00%) |  |
|  | 4 | 0 (0.00%) | 0 (0.00%) |  | 0 (0.00%) | 0 (0.00%) |  |
| **Echogenicity Flexor Digitorum Profundus** | 1 | 12 (85.70%) | 6 (60.00%) | 0.272 | 1 (20.00%) | 5 (100.00%) | **0.022** |
|  | 2 | 2 (14.30%) | 3 (30.00%) |  | 3 (60.00%) | 0 (0.00%) |  |
|  | 3 | 0 (0.00%) | 1 (10.00%) |  | 1 (20.00%) | 0 (0.00%) |  |
|  | 4 | 0 (0.00%) | 0 (0.00%) |  | 0 (0.00%) | 0 (0.00%) |  |
| **Echogenicity Flexor Carpi Ulnaris** | 1 | 13 (92.90%) | 6 (60.00%) | 0.134 | 3 (60.00%) | 3 (60.00%) | 0.135 |
|  | 2 | 1 (7.10%) | 3 (30.00%) |  | 1 (20.00%) | 2 (40.00%) |  |
|  | 3 | 0 (0.00%) | 1 (10.00%) |  | 1 (20.00%) | 0 (0.00%) |  |
|  | 4 | 0 (0.00%) | 0 (0.00%) |  | 0 (0.00%) | 0 (0.00%) |  |
| **Echogenicity Tibialis Anterior** | 1 | 10 (71.40%) | 2 (20.00%) | **0.030** | 0 (0.00%) | 2 (40.00%) | **0.004** |
|  | 2 | 4 (28.60%) | 4 (40.00%) |  | 2 (40.00%) | 2 (40.00%) |  |
|  | 3 | 0 (0.00%) | 3 (30.00%) |  | 3 (60.00%) | 0 (0.00%) |  |
|  | 4 | 0 (0.00%) | 1 (10.00%) |  | 0 (0.00%) | 1 (20.00%) |  |
| **PD Deltoid** | 0 | 12 (85.70%) | 5 (50.00%) | 0.209 | 1 (20.00%) | 4 (80.00%) | 0.086 |
|  | 1 | 2 (14.30%) | 3 (30.00%) |  | 2 (40.00%) | 1 (20.00%) |  |
|  | 2 | 0 (0.00%) | 1 (10.00%) |  | 1 (20.00%) | 0 (0.00%) |  |
|  | 3 | 0 (0.00%) | 1 (10.00%) |  | 1 (20.00%) | 0 (0.00%) |  |
|  | 4 | 0 (0.0%) | 0 (0.0%) |  | 0 (0.0%) | 0 (0.00%) |  |
| **PD Vastus Lateralis** | 0 | 6 (42.90%) | 1 (10.00%) | 0.118 | 0 (0.00%) | 1 (20.00%) | 0.312 |
|  | 1 | 7 (50.00%) | 9 (90.00%) |  | 5 (100.00%) | 4 (80.00%) |  |
|  | 2 | 1 (7.10%) | 0 (0.00%) |  | 0 (0.00%) | 0 (0.00%) |  |
|  | 3 | 0 (0.00%) | 0 (0.00%) |  | 0 (0.00%) | 0 (0.00%) |  |
|  | 4 | 0 (0.00%) | 0 (0.00%) |  | 0 (0.00%) | 0 (0.00%) |  |
| **PD Flexor Digitorum Profundus** | 0 | 7 (50.00%) | 4 (40.00%) | 0.553 | 2 (40.00%) | 2 (40.00%) | 0.881 |
|  | 1 | 6 (42.90%) | 6 (60.00%) |  | 3 (60.00%) | 3 (60.00%) |  |
|  | 2 | 1 (7.10%) | 0 (0.00%) |  | 0 (0.00%) | 0 (0.00%) |  |
|  | 3 | 0 (0.00%) | 0 (0.00%) |  | 0 (0.00%) | 0 (0.00%) |  |
|  | 4 | 0 (0.00%) | 0 (0.00%) |  | 0 (0.00%) | 0 (0.00%) |  |
| **PD Flexor Carpi Ulnaris** | 0 | 5 (35.70%) | 4 (40.00%) | 0.849 | 3 (60.00%) | 1 (20.00%) | 0.775 |
|  | 1 | 7 (50.00%) | 5 (50.00%) |  | 2 (40.00%) | 3 (60.00%) |  |
|  | 2 | 1 (7.10%) | 1 (10.00%) |  | 0 (0.00%) | 1 (20.00%) |  |
|  | 3 | 0 (0.00%) | 0 (0.00%) |  | 0 (0.00%) | 0 (0.00%) |  |
|  | 4 | 1 (7.10%) | 0 (0.00%) |  | 0 (0.00%) | 0 (0.00%) |  |
| **PD Tibialis Anterior** | 0 | 7 (50.0%) | 3 (30.0%) | 0.301 | 2 (40.0%) | 1 (20.0%) | 0.518 |
|  | 1 | 4 (28.6%) | 6 (60.0%) |  | 3 (60.0%) | 3 (60.0%) |  |
|  | 2 | 3 (21.4%) | 1 (10.0%) |  | 0 (0.0%) | 1 (20.0%) |  |
|  | 3 | 0 (0.0%) | 0 (0.0%) |  | 0 (0.0%) | 0 (0.0%) |  |
|  | 4 | 0 (0.0%) | 0 (0.0%) |  | 0 (0.0%) | 0 (0.0%) |  |
|  |  |  |  |  |  |  |  |

IIM: idiopathic inflammatory myopathies, HC: healthy controls, IBM: Inclusion Body Myositis, NAM: Necrotising Autoimmune Myopathy, PD: power Doppler, Statistically significant p<0.05, n: number of patients, SD: Standard deviation
