# Supplementary figures and images for: Muscle shear wave elastography, conventional B mode and power doppler ultrasonography in healthy adults and patients with autoimmune inflammatory myopathies: a pilot cross-sectional study
Source: BMC Musculoskelet Disord. 2021 Jun 12;22:537. doi: 10.1186/s12891-021-04424-0 (PMC8199828; doi:10.1186/s12891-021-04424-0)

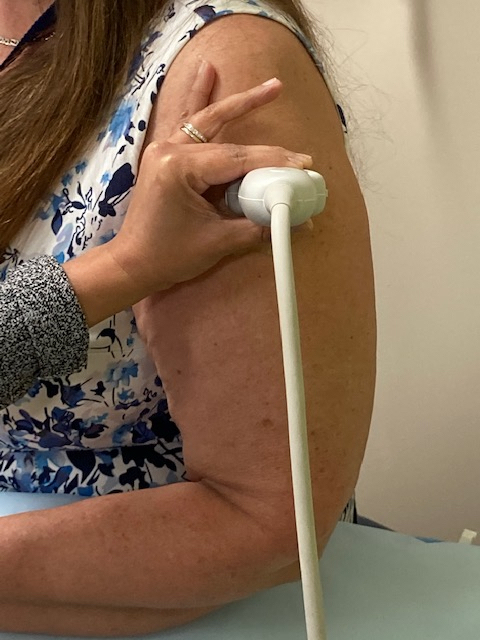

Supplement: Supplementary file 4 — Additional file 4: Supplementary Figure 1: The Deltoid probe position was 1/3 of the distance from the acromion to the lateral epicondyle. [file 12891_2021_4424_MOESM4_ESM.jpg]

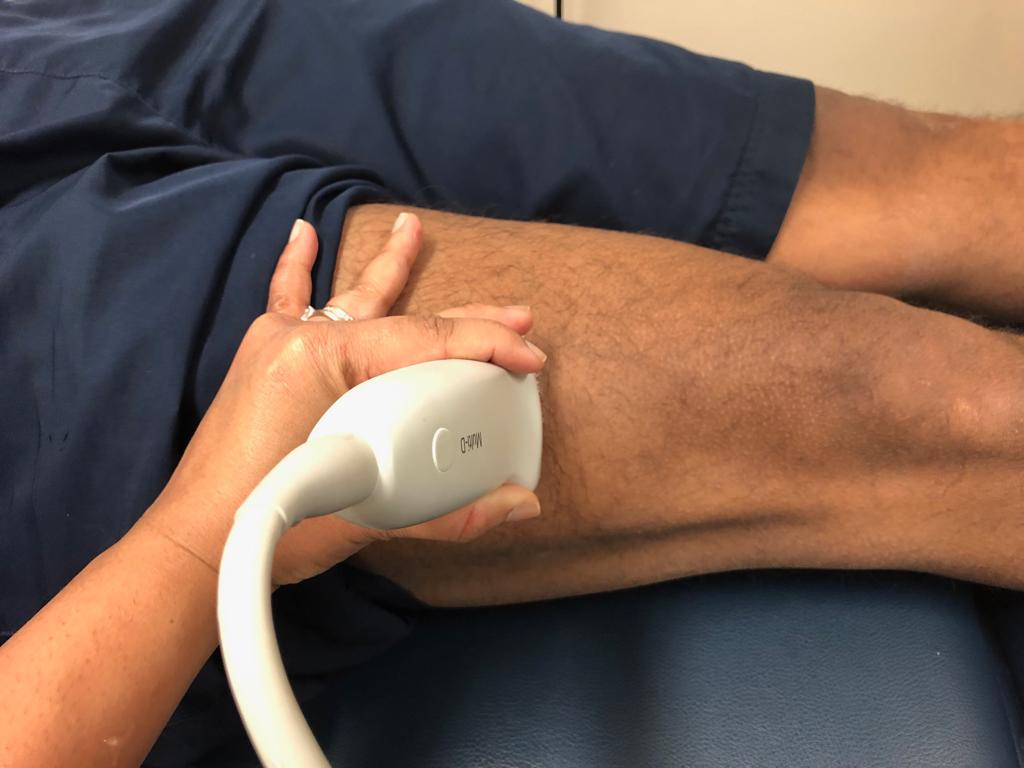

Supplement: Supplementary file 5 — Additional file 5: Supplementary Figure 2: The Vastus Lateralis probe position was ¼ of the distance from the anterior superior iliac spine (ASIS) to the superior border of the patella. [file 12891_2021_4424_MOESM5_ESM.jpg]

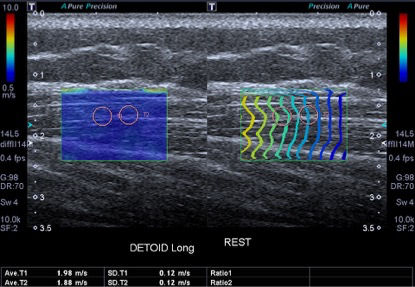

Supplement: Supplementary file 6 — Additional file 6: Supplementary Figure 3: Shear wave speed in the deltoid of a 28-year-old healthy female at rest showing the colour blue, which indicates lower muscle stiffness. [file 12891_2021_4424_MOESM6_ESM.jpg]

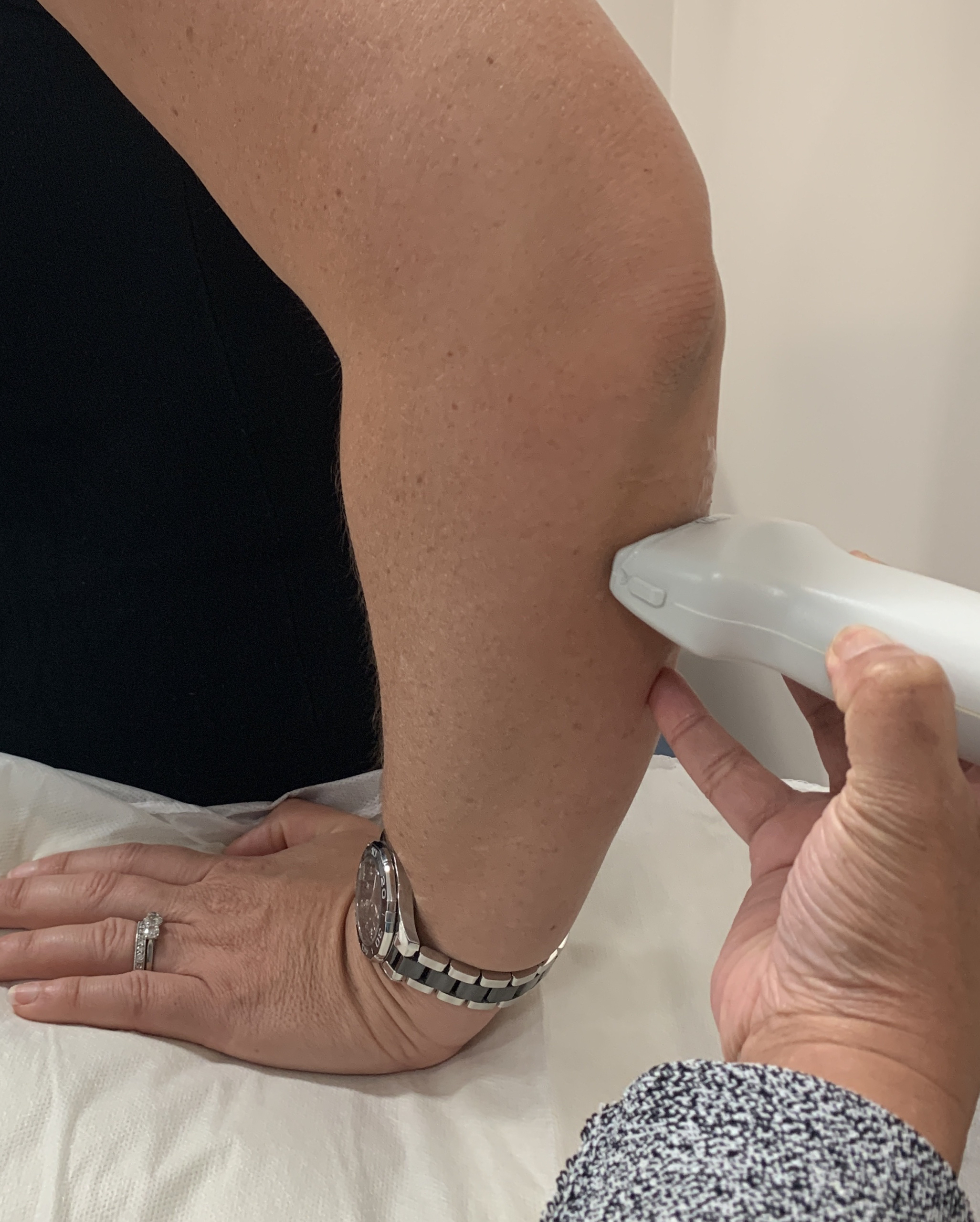

Supplement: Supplementary file 7 — Additional file 7: Supplementary Figure 4: The Flexor Digitorium Profundus and Flexor Carpi Ulnaris probe position was 5 cm from olecranon process. [file 12891_2021_4424_MOESM7_ESM.jpg]

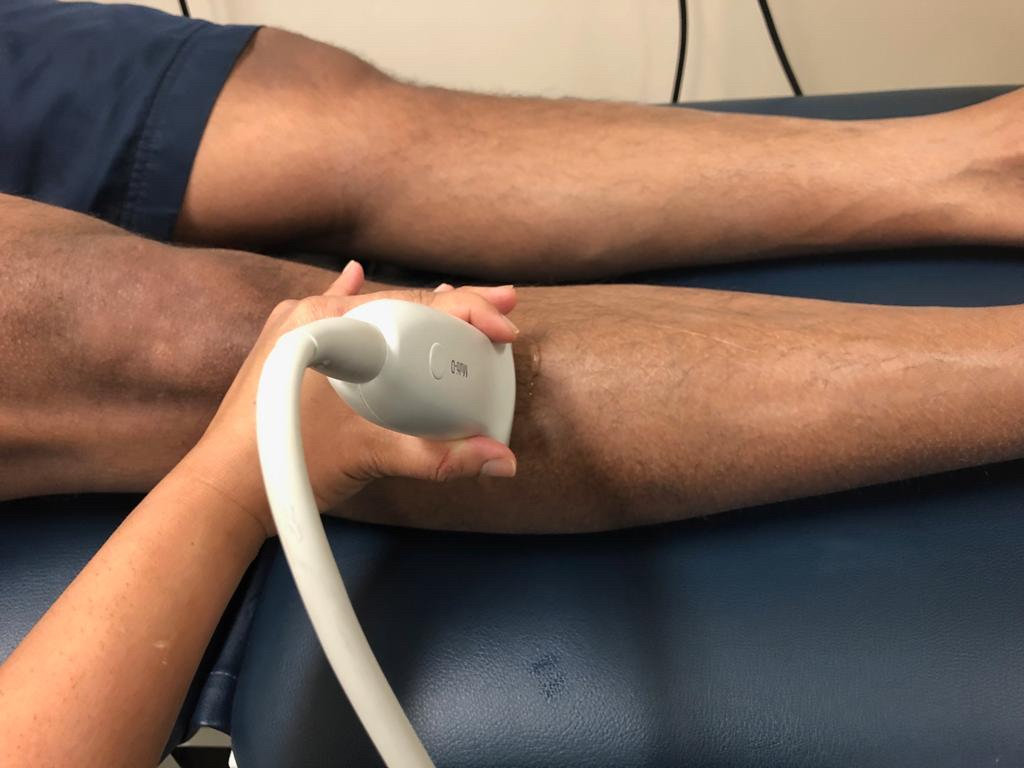

Supplement: Supplementary file 8 — Additional file 8: Supplementary Figure 5: The Tibialis Anterior muscle probe position is anterior shin just proximal to the calf bulk. [file 12891_2021_4424_MOESM8_ESM.jpg]
